# Longitudinal bidirectional relations between children’s negative affectivity and maternal emotion expressivity

**DOI:** 10.3389/fpsyg.2022.983435

**Published:** 2022-10-20

**Authors:** Lin Tan, Cynthia L. Smith

**Affiliations:** ^1^Department of Health Behavior and Health Systems, University of North Texas Health Science Center, Fort Worth, TX, United States; ^2^Department of Human Development and Family Science, Virginia Tech, Blacksburg, VA, United States

**Keywords:** negative affectivity, maternal positive expressivity, maternal negative expressivity, temperament, childhood

## Abstract

Although children’s negative affectivity is a temperamental characteristic that is biologically based, it is framed within and shaped by their emotional environments which are partly created by maternal emotion expressivity in the family. Children, in turn, play a role in shaping their family emotional context, which could lead to changes in mothers’ emotion expressivity in the family. However, these theorized longitudinal bidirectional relations between child negative affectivity and maternal positive and negative expressivity have not been studied from toddlerhood to early school-age. The current study utilized a cross-lagged panel model to examine the reciprocal relations between children’s negative affectivity and maternal expressivity within the family over the course of early childhood. Participants were 140 mother–child dyads (72 boys, mean age = 2.67 years, primarily White). Mothers reported the positive and negative expressivity in the family and children’s negative affectivity in toddlerhood (T1), preschool (T2), and school-age (T3). Maternal negative expressivity and child negative affectivity at T1 were significantly correlated. Maternal negative expressivity at T1 significantly predicted child negative affectivity at T3. Children’s negative affectivity at T2 significantly predicted mothers’ negative expressivity at T3. Mothers’ positive expressivity was not related to children’s negative affectivity at any of the three time points. The findings demonstrate the reciprocal relations between children’s negative affectivity and maternal negative expressivity in the family, suggesting the importance of the interplay between child temperament and maternal expressivity within the family emotional context.

## Introduction

Temperament is defined as biologically-based individual differences in emotional reactivity and self-regulation influenced by heredity, environment, and experience (Rothbart and Derryberry, 1981; Rothbart et al., 2004). Negative affectivity, which refers to the tendency to become negatively aroused and reactive to stimuli (Rothbart et al., 2001), is an aspect of temperament that has been found to be related to anxiety and depression symptoms (Lonigan et al., 2003; van der Bruggen et al., 2010), lower social competence (Sallquist et al., 2009), and internalizing and externalizing behaviors (Engle and McElwain, 2011). Although emotional reactivity is biologically based, it is refined within children’s environments, particularly the family, from the beginning of development. Therefore, it is important to examine the factors within the family that are related to the changes and development of children’s negative affectivity over time.

Previous studies have established parenting behaviors, including psychological control, rejection, parenting efficacy, and harsh discipline (e.g., van der Bruggen et al., 2010; Troutman et al., 2012; Xing et al., 2017; Diaz et al., 2019), as major contributors in determining the development of negative affectivity in young children. These parenting behaviors are often considered in the goodness-of-fit between children’s temperament and their environment (Thomas and Chess, 1977), where the expectation is that children have more optimal outcomes when parenting behaviors are in accord with their temperament. However, equally important but much less studied is the emotion expressivity in the family context. Mothers’ emotion expressivity – a persistent style in displaying verbal and nonverbal expressions toward other family members (Halberstadt et al., 1995) – contributes to the quality of the family emotional climate (Halberstadt et al., 1999), and thus the current study focused on how mothers’ emotion expressivity within the family context and child negative affectivity relate to each other.

Children can directly observe and imitate mothers’ emotional reactions to common situations in the family and toward family members, such as expressing gratitude for a favor or voicing anger over a mistake. For children, these emotional reactions may serve as foundational models to how emotions are supposed to be expressed (i.e., emotion display rules). For example, when mothers express frequent and intense negative emotions, in response to mundane situations (e.g., threatening a family member or being angry at a family member’s small mistake), children with high negative affectivity (i.e., those with a natural predisposition to react with negative emotions), may think that it is perfectly normal to express these negative emotions regularly or strongly. On the other hand, children with mothers who habitually express more positive and less negative emotions in the family may consider responding to situations with negative emotions to be atypical, which prompts them to learn to down-regulate their negative reactions.

In addition, during mother–child interactions, mothers’ emotions are directly expressed toward their children. Exposure to high levels of negative emotions expressed by mothers may lead to over-arousal in children, especially children with naturally high negative affectivity (Eisenberg et al., 2001). High levels of negative expressivity may adversely affect the quality and security of mother–child relationships, precluding mothers from adequately teaching or supporting the development of their children’s emotion regulation (Valiente et al., 2004b). These children, thus, may not have opportunities or be supported to learn the skills needed to regulate their emotional reactivity. Similarly, when more positive emotions are expressed toward children, it could provide emotional support to them so that they are better able to regulate their own negative emotions when distressed (Garner, 1995; Eisenberg et al., 2001; Fredrickson, 2001). Many studies have shown that maternal emotion expressivity is related to children’s effortful control, emotion regulation, social competence, and problem behaviors (Valiente et al., 2006; McCoy and Raver, 2011; Miller et al., 2015; Are and Shaffer, 2016; Tan and Smith, 2019), and hence more surprising that the empirical evidence for the association between maternal expressivity and child negative affectivity is sparse.

Indeed, the few studies that have investigated this association between maternal expressivity and child negativity found a significant correlation between them. For example, maternal negative expressivity was found to significantly predict children’s negative expressions when watching a distressing film in a sample of 7-year-olds (Valiente et al., 2004a). Higher maternal positive and lower negative affective displays during family interactions were significantly related lower adolescent negative affectivity (Davenport et al., 2011). One study, however, showed that parental expressivity was not significantly associated with children’s temperamental anger/frustration or sadness in a sample of 4.5-to 8-year-olds (Wang et al., 2016). These mixed findings may be due to the cross-sectional analyses of these constructs. Furthermore, the ages of the samples of these previous studies were middle to late childhood or early adolescence, instead of early childhood. The longitudinal design of the current study allows us to better capture the changes in the relations between child negative reactivity and maternal expressivity from toddlerhood to early school-age. This developmental period is especially important to study because children spend more time in the family in early childhood and early school years than they do in later school years and adolescence. Toddlers often depend on their caregivers to co-regulate their emotions and behaviors and help them learn more advanced forms of emotion regulation strategies and behaviors (Kopp, 1982), and thus mothers’ socialization of their children’s emotion expression is critical during early childhood, while the executive systems of children undergo rapid development (Zelazo and Carlson, 2012; Fay-Stammbach et al., 2014).

Not only may mothers’ emotion expressivity affect children’s negative affectivity, but children likely play a role in shaping their family emotional context (Cole and Tan, 2007), which could lead to changes in mothers’ emotion expressivity. Negative affectivity, including anger/frustration, sadness, and fear, is considered to be a key aspect of difficult temperament (Bates, 1989; Shiner, 1998). Children high in negative affectivity express frequent and intense negative emotions to stressors and have difficulty adapting (Bates and Pettit, 2007), which may elicit less supportive maternal socialization (van der Bruggen et al., 2010). For example, 3-year-olds’ negative expressivity was related to a higher likelihood of mothers expressing more negative emotions when children were 4 years of age (Nelson et al., 2012). Therefore, the relations between mothers’ emotional expressivity and children’s negative affectivity are likely to be bidirectional. Similar to the process described in the coercive cycles (Patterson, 1982; Scaramella and Leve, 2004), children, who are high in negative affectivity, are highly reactive and express more negative emotions. These negative emotions potentially create distress and disruptions in the family, which evokes more negative and less positive emotions and expressions in their mothers. These maternal emotional expressions then create more stressful emotional contexts for children that amplify negative affectivity in children. Through this process of mutual reinforcement, cycles of negativity, or negative mother–child reciprocities, can be initiated and are detrimental for both parties.

These reciprocal relations between children’s negative reactivity and maternal emotion expressivity in the family are important to understanding how temperament intersects with the environment to shape children’s emotional development over time. Thus, the current study utilized a cross-lagged panel model to examine the bidirectional relations between children’s negative affectivity and mothers’ positive and negative expressivity in the family throughout early childhood. We hypothesized that higher levels of maternal positive expressivity and lower levels of maternal negative expressivity would predict lower levels of child negative affectivity over time, and reciprocally higher levels of child negative affectivity would predict lower levels of maternal positive expressivity and higher levels of maternal negative expressivity over time.

## Materials and methods

### Participants

Children and their mothers in the Mid-Atlantic region of the United States participated in a longitudinal study following children from toddlerhood to school-age from 2005 to 2012. At toddlerhood (T1), 140 mothers and children (72 boys, *M* = 2.67 years, *SD* = 0.13) participated; 116 mothers and children (62 boys, *M* = 4.91 years, *SD* = 0.30) participated again at preschool (T2); 109 mothers (60 boys, *M* = 8.80 years, *SD* = 0.42) completed the questionnaires at school-age (T3). At T1, the majority of mothers, 96.4%, were married or living with their children’s father. The average family income was 4.44 on a 7-point scale where 1 = less than $15,000, 4 = $45,000–$60,000, and 7 = more than $100,000. Most mothers, 71.4%, had a college degree or higher, and the majority of mothers, 95.7%, were European American. The families remaining at T3 did not differ significantly from the families who discontinued participation on children’s age and sex, maternal report of fathers’ age and race/ethnicity, family income, child negative affectivity, and maternal positive and negative expressivity at T1. Mothers were older, *M* = 33.32, *SD* = 4.43, in the families who continued participation compared to those who did not, *M* = 31.03, *SD* = 4.15, *t*(134) = −2.50, *p* = 0.01. Families who continued participation had more White mothers, 98.2%, than those who discontinued participation, 86.7%, Fisher’s exact = 0.02, *p* = 0.02.

### Procedures

Mothers were recruited to participate in a study when their children were between the ages of 30 and 36 months. At T1, approximately half of the families (51.4%) had participated in a previous study in a different lab and were contacted about participating in the current study. The other half of the families (48.6%) were recruited by placing fliers in places where families of young children would often go (e.g., story time at the local library) and by asking childcare centers to give fliers to families with children between 30 and 36 months of age (T1). Mothers were contacted about a follow-up assessment when children were 4–5 years of age (T2) and again when children were 8–9 years of age (T3). At each assessment, mothers completed several questionnaires, including a demographic form and the Self-Expressiveness in the Family Questionnaire (SEFQ; Halberstadt et al., 1995). At T1, mothers completed the Early Childhood Behavior Questionnaire (ECBQ; Putnam et al., 2006). At T2, mothers completed the Child Behavior Questionnaire – Short Form (CBQ–SF; Putnam and Rothbart, 2006), and they completed the Temperament in Middle Childhood Questionnaire (TMCQ; Simonds and Rothbart, 2004) at T3.

### Measures

#### Maternal emotion expressivity

Mothers reported their positive and negative emotion expressivity in the family on a 9-point scale (1 = rarely express these feelings to 9 = frequently express these feelings) at all three time points. The positive (23 items, *α*s = 0.88, 0.90, 0.93, e.g., “Expressing deep affection or love for someone.”) and negative (17 items, *α*s = 0.86, 0.87, 0.87, e.g., “Showing how upset you are after a bad day.”) subscales from the SEFQ were used. Composite scores of positive and negative expressivity were created by averaging the items in each subscale.

#### Child negative affectivity

To assess child negative affectivity, mothers rated their children’s temperamental frustration/anger, sadness, and fear at all three time points. At T1, mothers completed the frustration/anger subscale (12 items, *α* = 0.84, e.g., “When s/he could not find something to play with, how often did your child get angry?”), sadness subscale (12 items, *α* = 0.83, e.g., “When told “no,” how often did your child become sadly tearful?”), and fear subscale (11 items, *α* = 0.76, e.g., “During everyday activities, how often did your child startle at loud noises (such as a fire engine siren)?”) from the ECBQ on a 7-point scale (1 = extremely untrue of my child to 7 = extremely true of my child). At T2, the frustration/anger subscale (six items, *α* = 0.78, e.g., “Gets quite frustrated when prevented from doing something s/he wants to do.”), sadness subscale (seven items, *α* = 0.62, e.g., “Tends to become sad if the family’s plans do not work out.”), and fear subscale (six items, *α* = 0.60, e.g., “Is afraid of loud noises.”) from the CBQ–SF was used; mothers rated items on a 7-point scale (1 = extremely untrue of my child to 7 = extremely true of my child). At T3, mothers completed the anger/frustration subscale (seven items, *α* = 0.85, e.g., “Gets angry when s/he cannot find something s/he is looking for.”), sadness subscale (10 items, *α* = 0.82, e.g., “Tends to become sad if plans do not work out.”), and fear subscale (nine items, *α* = 0.80, e.g., “Is afraid of loud noises.”) from the TMCQ on a 5-point scale (1 = almost always untrue to 5 = almost always true). Composite scores of child negative affectivity were computed by averaging scores from the frustration/anger, sadness, and fear subscales at each time point.

## Results

Means, standard deviations, and correlations among the study variables are shown in Table 1. Maternal positive expressivity measures at all three time points were significantly correlated with each other as were maternal negative expressivity and child negative affectivity. Maternal negative expressivity and child negative affectivity were significantly correlated to each other within each time point. Maternal positive expressivity was not significantly correlated with maternal negative expressivity or child negative affectivity at any of the three time points. Mothers’ age was significantly correlated with child negative affectivity at T2, *r* = −0.19, *p* = 0.04; mothers’ education was significantly correlated with mothers’ negative expressivity, *r* = −0.20, *p* = 0.03 and child negative affectivity, *r* = −0.24, *p* = 0.01, at T3, so we controlled for them in the cross-lagged longitudinal analysis. Partial correlations controlling for mothers’ age and education are also presented in Table 1. Child age, child sex, and maternal race/ethnicity were not significantly related to any of the study variables, and thus they were not controlled for in the following analysis.

**Table 1:** Means, standard deviations, and correlations among study variables.

	1	2	3	4	5	6	7	8	9
T1									
1. Maternal positive expressivity	–	0.16	0.06	0.70^*^	−0.05	0.03	0.70^*^	0.01	0.05
2. Maternal negative expressivity	0.11	–	0.31^*^	0.21^*^	0.56^*^	0.25^*^	0.10	0.58^*^	0.32^*^
3. Child negative affectivity	−0.03	0.35^*^	–	0.11	0.21^*^	0.55^*^	0.05	0.31^*^	0.53^*^
T2									
4. Maternal positive expressivity	0.69^*^	0.18	0.04	–	0.20	0.04	0.75^*^	0.18	0.06
5. Maternal negative expressivity	−0.09	0.54^*^	0.19^*^	0.19^*^	–	0.29^*^	0.03	0.73^*^	0.23^*^
6. Child negative affectivity	−0.03	0.33^*^	0.58^*^	0.01	0.28^*^	–	0.10	0.44^*^	0.64^*^
T3									
7. Maternal positive expressivity	0.70^*^	0.11	0.00	0.71^*^	0.04	0.07	–	0.10	0.08
8. Maternal negative expressivity	0.02	0.58^*^	0.35^*^	0.15	0.71^*^	0.44^*^	0.10	–	0.41^*^
9. Child negative affectivity	0.00	0.34^*^	0.55^*^	0.05	0.24^*^	0.64^*^	0.05	0.44^*^	–
*M*	7.06	4.15	3.24	7.16	4.11	3.51	7.05	3.99	2.58
SD	0.85	1.07	0.63	0.87	1.08	0.74	0.86	1.04	0.56

^*^p < 0.05. Partial correlations controlling for maternal age and education are presented above the diagonal.

**Figure 1:**
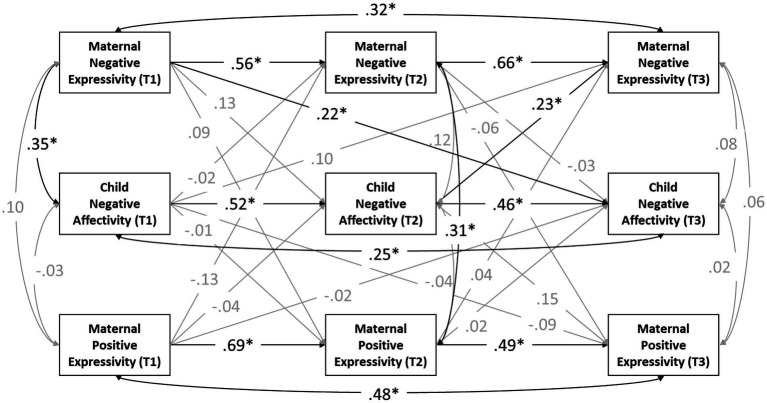
Reciprocal relations among child negative affectivity and maternal positive and negative expressivity within the family. Standardized coefficients are presented. Black lines represent significant paths, with gray lines representing non-significant paths. Mothers’ age and education were controlled for in the model and for ease of presentation, the paths are not presented. ^*^p < 0.05.

To examine the bidirectional relations of maternal emotion expressivity and child negative affectivity, we conducted a cross-lagged longitudinal analysis with MPlus 8.7 using maximum likelihood parameter estimates with robust standard errors (MLR). For model fit indices, we considered RMSEA ≤ 0.08, CFI ≥ 0.90, and SRMR ≤ 0.08 to be adequate fit (Vandenberg and Lance, 2000; Marsh et al., 2004). The results from the cross-lagged longitudinal model, *χ*^2^(2) = 0.14, *p* = 0.93, RMSEA = 0.00, CFI = 1.00, SRMR = 0.00, are shown in Figure 1. Mothers’ negative expressivity and child negative affectivity were significantly correlated at T1. Mothers’ negative expressivity at T1 significantly predicted children’s negative affectivity at T3. Child negative affectivity at T2 was significantly associated with mothers’ negative expressivity at T3. Mothers’ positive expressivity was not significantly related to children’s negative affectivity.

## Discussion

The current study investigated the reciprocal relations between maternal emotion expressivity and child negative affectivity across three time points from toddlerhood to early school-age. Although child negative affectivity was modestly stable across the three time points, changes in it over time were related to mothers’ negative expressivity in the family. Similarly, the changes in mothers’ negative expressivity were associated with child negative affectivity. The findings demonstrate bidirectional relations between children’s negative affectivity and mothers’ negative expressivity in the family, suggesting the importance of the interplay between child temperament and mothers’ expressivity in the family.

Adding to a rich body of literature demonstrating bidirectional relations between child temperament and specific parenting behaviors (e.g., Lengua and Kovacs, 2005; Lee et al., 2012; Klein et al., 2018; Wittig and Rodriguez, 2019), our novel results show higher maternal negative expressivity within the family at toddlerhood predicted higher child negative affectivity at school-age. Higher child negative affectivity at preschool-age predicted higher negative expressivity of the mothers at school-age. Higher maternal negative expressivity and child negative affectivity were correlated at toddlerhood, and the relations between maternal negative expressivity and child negative affectivity may be partially due to shared heritable influences (Boivin et al., 2005; Liu et al., 2020). In addition, mothers’ and children’s negative expressions may influence each other through emotional contagion (Butler, 2015). For example, by simply being close to their mothers who were having a stress response after a high arousal negative task, infants displayed similar physiological responses (Waters et al., 2017). The findings in our data that maternal negative expressivity in the family and child negative affectivity were correlated in toddlerhood may be explained by emotional contagion, modeling, and socialization, likely due to the great amount of time mothers and their children share together in toddlerhood. To further investigate how negativity of mothers and children influence each other, we encourage future research to take maternal expressivity specifically to the child into consideration to examine how it relates the quality of mother–child interactions.

Our results supported bidirectional relations between child negative affectivity and maternal negative expressivity. Mothers’ high negative expressivity in the family may result in an emotionally stressful environment and a poor affective quality of mother–child interactions, which prevents children from being supported to learn emotion regulation skills necessary to control their negative reactivity. Over time, negative mother–child exchanges may lead to greater neural responses to negative emotional information and increased negative reactivity in children (Tan et al., 2020). On the other hand, increased child negative reactivity may elicit more distress reactions from mothers, which may result in mothers expressing more negative emotions within the family. For example, children high in negative affectivity tend to display more emotionally dysregulated behaviors during mother–child interactions and contribute to high maternal distress (Pesonen et al., 2008; Yap et al., 2011).

Although our results supported the bidirectional relations between maternal negative expressivity and child negative affectivity, we do not have evidence for vicious cycles of negativity across early childhood. The findings that child negative affectivity and maternal negative expressivity at school-age but not preschool-age were predicted by earlier maternal negative expressivity and child negative affectivity, respectively, is interesting. It is possible that after the transition to school, children spend considerably less time at home – the environment children are familiar with – and have to face changes and new stressors at school, such as increased peer interactions and pressure from school work. Thus, the risks associated with their negative affectivity became more salient during the school-age period than prior developmental periods, although the early association of negativity between mothers and children in toddlerhood may potentially serve as a basis for the long-term patterns found in this work.

Our findings are consistent with the coercion model which suggests that the significant impact of coercive cycles of parent–child interactions may not become obvious until after children enter school (Scaramella and Leve, 2004). Because mothers’ negative expressivity in toddlerhood appears to have a long-term influence on child negative affectivity, it is important for practitioners to intervene early by teaching mothers strategies to manage their negative emotions to prevent the long-term adverse impact of maternal negative expressivity on children. At the same time, because children’s negative reactivity during preschool years may also have an impact on mothers, it is equally important to provide resources and support for children to learn emotion regulation skills to help them regulate and utilize their negative emotions adaptively (Tan and Smith, 2018; Tan et al., 2022). Researchers, practitioners, and schools may consider a family-based program that targets both the parents and the children to help them create a more optimal family emotional climate. Because children’s self-regulation develops rapidly across early childhood (Kopp, 1982; Montroy et al., 2016) and it likely plays a critical role in understanding the relations between child negative affectivity and maternal negative expressivity (Bariola et al., 2011), future research should consider examining how children’s and parents’ self-regulation characteristics, such as effortful control, influence the longitudinal relations between child negative affectivity and maternal negative expressivity.

Unexpectedly, maternal positive expressivity was not significantly related to child negative affectivity, which indicates that positive and negative expressivity may be uniquely associated with child temperament. A previous study found that mothers’ positive affect during a play session was associated with child positive affect but not child negative affect, whereas maternal negative affect was related to both child positive and negative affect (Isley et al., 1999). It is also possible that maternal positive expressivity provides a supportive emotional environment for the development of effortful control (Eisenberg et al., 2001), which is related to the regulation of negative emotions, but maternal positive expressivity is not directly associated with child negative affectivity. In addition, the interaction of maternal positive and negative expressivity and how it may impact the goodness-of-fit between mothers and children may be another potential reason why positive expressivity was not related to child negative affectivity. Specifically, emotionally expressive mothers who are high in positive expressivity might express higher levels of negative emotions too. Compared to mothers who were more positive and less negative, emotionally expressive mothers could create a high-arousal emotional climate in the family, which might be particularly stressful for children high in negative affectivity. Future research should investigate how the interaction of maternal positive and negative expressivity would relate to child negative affectivity.

### Limitations and future directions

The current study extends the existing literature by examining the bidirectional relations between maternal emotion expressivity and child negative affectivity across three time points. The results provide evidence for bidirectional relations between maternal negative expressivity and child negative affectivity in early childhood. However, the findings of the current study should be interpreted with the following limitations in mind. First, the majority of the sample was middle-class, highly educated, European American two-parent mother–father families. Thus, the findings should be replicated by future studies using samples from other cultures, ethnicities, or socioeconomic backgrounds or samples that have diverse family characteristics. Additionally, all constructs of interest in the current study were measured through maternal report. Although mothers’ ratings of child temperament and their own expressivity within the family are reliable and valid measures (Halberstadt et al., 1995; Rothbart et al., 2001), they may have common method bias, which could potentially lead to an inflation of relations among the study variables. Future research investigating the reciprocal relations between child temperament and maternal expressivity should consider using multiple methods, such as observations and multiple reporters. We also focused on a global assessment of maternal expressivity in the family in the current study without considering maternal expressivity specifically to the child. Shared genetic factors also may partially explain the associations between maternal negative expressivity and child negative affectivity. Future studies utilizing other research designs, especially adoption designs (Liu et al., 2020), could further disentangle the genetic and environmental factors contributing to the relations between maternal negative expressivity and child negative affectivity.

Family stressors, such as job loss, may play an important role in the changes in mothers’ positive and negative expressivity. How these contextual factors along with other sources of stress and support influence mothers’ expressivity is an important research question to be studied. Finally, the emotional context within a family is created by all family members. Because of our study design, we were only able to focus on the emotional dynamics between the mother and one child. Other caregivers and siblings within the family also make an important contribution to the family emotional climate (e.g., Volling et al., 2002; Modry-Mandell et al., 2007; Yaremych and Volling, 2020; MacNeill et al., 2021), so we encourage researchers to continue extending knowledge on how family emotional contexts and child temperament influence each other by including multiple family members. In sum, our findings advance the knowledge of and provide empirical evidence for bidirectional relations between maternal emotion expressivity and child negative affectivity across early childhood.

## Data availability statement

The raw data supporting the conclusions of this article will be made available by the authors, without undue reservation.

## Ethics statement

The studies involving human participants were reviewed and approved by Virginia Tech Institutional Review Board. Written informed consent to participate in this study was provided by the participants’ legal guardian/next of kin.

## Author contributions

LT and CLS contributed to the conception and design of the current study and manuscript writing and revision. CLS was responsible for protocol design, data collection, project administration, and funding acquisition. LT performed the statistical analysis and wrote the first draft of the manuscript. All authors contributed to the article and approved the submitted version.

## Funding

This research was supported by funds from a Virginia Tech ASPIRES (A Support Program for Innovative Research Strategies) Award, a Virginia Tech College of Liberal Arts and Human Sciences Jerome Niles Faculty Research Award, a Virginia Tech College of Liberal Arts and Human Sciences Faculty Fellowship Award, and the Virginia Tech Institute for Society, Culture & Environment. All funds were awarded to CLS.

## Conflict of interest

The authors declare that the research was conducted in the absence of any commercial or financial relationships that could be construed as a potential conflict of interest.

## Publisher’s note

All claims expressed in this article are solely those of the authors and do not necessarily represent those of their affiliated organizations, or those of the publisher, the editors and the reviewers. Any product that may be evaluated in this article, or claim that may be made by its manufacturer, is not guaranteed or endorsed by the publisher.
